# Functional and Radiological Outcomes of Calcar Buttress Biplane Double-Supported Screw Fixation (BDSF) Using a Locking Compression Screw System for Intracapsular Femoral Neck Fractures: A Prospective Observational Study

**DOI:** 10.7759/cureus.110855

**Published:** 2026-06-14

**Authors:** Mohamed P Azlam, Ravi Sharma, Vishal Kumar, Jayant Kothale

**Affiliations:** 1 Orthopaedics, Government Medical College - Seoni, Seoni, IND; 2 Orthopaedics, Netaji Subhash Chandra Bose (NSCB) Medical College and Hospital - Jabalpur, Jabalpur, IND; 3 Orthopaedics, Netaji Subhas Medical College and Hospital - Bihta, Patna, IND

**Keywords:** functional and radiological outcome, functional outcomes, intracapsular femoral neck fractures, operative time, pain on vas

## Abstract

Background

Intracapsular femoral neck fractures in adults aged 40-60 years present a therapeutic challenge. Calcar buttress fixation using the biplane double-supported screw fixation (BDSF) concept serves to enhance load transfer through dual cortical support. This study aimed to evaluate the functional and radiological outcomes of calcar buttress BDSF fixation using a locking compression screw system in adults aged 40-60 years with intracapsular femoral neck fractures.

Methods

This prospective observational study was conducted at a tertiary care hospital from September 2022 to June 2024 and included adults aged 40-60 years with intracapsular femoral neck fractures presenting within three weeks of injury. All 25 patients underwent closed reduction and internal fixation using calcar buttress BDSF with a titanium locking compression screw system. Standardized rehabilitation included progressive weight-bearing from toe-touch at one week to full weight-bearing by 8-12 weeks. Patients were assessed at 1, 3, 6, and 12 months using the Harris Hip Score (HHS) and Visual Analogue Scale (VAS). Radiological union, Pauwels type-wise outcomes, and complications were recorded. Serial functional outcomes were compared using paired t-tests, and subgroup categorical comparisons were analyzed using Fisher's exact test, as appropriate.

Results

Mean age was 50.6 years (range 40-60 years); 16 of 25 patients (64.0%) were male. Falls accounted for 16 of 25 injuries (64.0%). Mean operative time was 58.8 minutes, with a mean intraoperative blood loss of 44 mL. Mean HHS improved significantly from 72.72 ± 7.22 at one month to 87.84 ± 7.30 at 12 months (p < 0.001). At 12 months, 14 of 25 patients (56.0%) achieved excellent outcomes, 7 of 25 (28.0%) achieved good outcomes, and 4 of 25 (16.0%) achieved fair outcomes; none had poor results. Mean VAS decreased from 8.04 ± 0.74 to 2.68 ± 1.38 (p < 0.001). Overall radiological union at 12 months was achieved in 24 of 25 patients (96.0%; 95% CI: 80.5%-99.3%). Union rates by Pauwels type were as follows: type 1, 4/4 (100.0%; 95% CI: 51.0%-100.0%); type 2, 11/12 (91.7%; 95% CI: 64.6%-98.5%); and type 3, 9/9 (100.0%; 95% CI: 70.1%-100.0%). Mean femoral neck shortening at 12 months was 5.3 ± 1.4 mm overall and was greatest in Pauwels type 3 fractures (6.5 ± 1.0 mm; p < 0.001).

Conclusions

Calcar-buttress BDSF fixation using a locking compression screw system may be considered a head-preserving fixation technique with encouraging early results for intracapsular femoral neck fractures in adults aged 40-60 years; however, the modest sample size and wide subgroup confidence intervals require cautious interpretation and preclude definitive comparative conclusions. Larger comparative studies are needed to confirm these findings.

## Introduction

Intracapsular femoral neck fractures constitute approximately half of all hip fractures, with projected global increases from 1.66 million in 1990 to 6.26 million by 2050 [1]. While arthroplasty is widely accepted for elderly patients, optimal management in younger adults (40-60 years) remains controversial [2]. We focused on this 40-60-year age group because it represents a borderline, skeletally mature but still functionally active cohort in whom surgeons must often choose between head-preserving fixation and arthroplasty. Preserving the native femoral head is desirable in this age group because arthroplasty is associated with higher long-term revision rates and activity limitations [3]. Conventional fixation with multiple cannulated cancellous screws (CCS) arranged in an inverted triangle offers shorter operative time and minimal blood loss but may fail to provide sufficient biomechanical stability, especially in osteoporotic bone or vertical fracture patterns [4]. In the present study, however, bone quality was not formally quantified, and this reference is made in the context of the broader fixation literature. Loss of interfragmentary compression and limited cortical support can lead to non-union, collapse, and avascular necrosis (AVN), with reported failure rates of 10%-30% [5].

To address these constraints, Filipov described the biplane double-supported screw fixation (BDSF) technique, which positions screws in two planes to achieve medial calcar and lateral diaphyseal cortical support, creating a beam-like configuration that better resists vertical loading and shear forces [6-9]. Clinical series using BDSF have reported favorable union rates exceeding 95%, though biomechanical studies comparing BDSF with plate-augmented constructs suggest continued evolution in fixation strategies [10,11].

The primary objective of this study was to evaluate functional and radiological outcomes of calcar buttress BDSF fixation using a locking compression screw system in adults aged 40-60 years with intracapsular femoral neck fractures. Secondary objectives were to assess Pauwels type-wise union and shortening outcomes, complications, and the association between femoral neck shortening and functional outcome.

## Materials and methods

Study design and setting

This prospective observational study was conducted in the Department of Orthopaedics, Netaji Subhash Chandra Bose Medical College and Hospital, Jabalpur, Madhya Pradesh, India, from September 2022 to June 2024. The study was approved by the Institutional Ethics Committee (IEC), Netaji Subhash Chandra Bose Medical College, Jabalpur (Approval No. IEC/2022/8629-132, dated September 05, 2022), prior to patient recruitment. Written informed consent was obtained from all participants before enrollment.

Sample size

An a priori sample size of 50 patients was calculated; however, due to mid-study unavailability of the specific locking compression screw system under the Ayushman Bharat Yojana reimbursement scheme, only 25 eligible patients were treated and included in the analysis.

Post-hoc power analysis was performed for the primary outcome (HHS improvement from 1 to 12 months). The observed mean difference was 15.12 points (SD of difference = 7.22), yielding a Cohen’s d of 2.09 - a very large effect size. Using a two-tailed paired t-test with α = 0.05 and n = 25, the achieved power was >99.9%, confirming that the primary outcome analysis was not underpowered despite the reduced sample. However, we acknowledge that the Pauwels type subgroup analyses (n = 4, n = 12, and n = 9) are underpowered for detecting differences in union rates, as noted in the limitations section.

Patient selection

Adults aged 40-60 years with intracapsular fracture of the femoral neck (subcapital, transcervical, or basicervical) presenting within three weeks of injury were included. This age range was selected to study a skeletally mature, physiologically active population in whom head-preserving fixation remains clinically relevant but arthroplasty is also a realistic alternative. Patients medically unfit for anesthesia or surgery, those with compound fractures associated with vascular injuries, those with associated pelvic injuries, and patients unwilling to undergo surgery were excluded from the study.

Preoperative evaluation

All patients underwent detailed clinical assessment, including general physical examination and local hip examination, along with baseline laboratory investigations. Radiographic evaluation comprised standard anteroposterior and lateral views of the pelvis and affected hip. Pauwels’ classification was used to categorize fractures based on fracture line inclination [12]. No formal bone mineral density assessment with DEXA, Singh index grading, or cortical thickness measurement was performed; bone quality was assessed only clinically and from standard radiographs as part of routine care. Computed tomography (CT) or magnetic resonance imaging (MRI) was not part of the routine imaging protocol and was reserved only for cases with diagnostic uncertainty or suspected pathological lesions; neither indication was present in this consecutive series.

Surgical technique

All surgeries were performed under spinal anesthesia on a fracture table with image intensifier guidance. All procedures were performed by a single senior consultant orthopaedic surgeon with more than 10 years of experience in hip fracture surgery and prior familiarity with the BDSF technique, ensuring consistency in surgical execution across the series. The initial four cases were supervised by an experienced mentor, after which the principal surgeon operated independently. Closed reduction was achieved using traction and gentle internal rotation (Whitman maneuver), with reduction adequacy assessed using the Garden alignment index, targeting 155-180 degrees on both views [13]. A 6-7 cm lateral incision was made at the inferior border of the greater trochanter, exposing the lateral periosteum of the proximal femoral diaphysis. A titanium locking compression screw system (6.5-7.5 mm) was employed in calcar buttress configuration. The distal guidewire was inserted 5-7 cm distal to the greater trochanter, directed at 150-165 degrees to engage the calcar and posterior femoral head. The middle guidewire was placed 2-4 cm proximal to the distal wire at 135-140 degrees, directed to contact the calcar and enter the anterior head. The proximal guidewire was inserted 1.5-2 cm anterior to the middle wire, also directed anteriorly. Following cannulated drilling, appropriate-length locking screws were inserted, with the proximal and middle screws tightened first to achieve interfragmentary compression, followed by the distal screw.

Rehabilitation protocol

Postoperatively, patients were positioned supine with a pillow under the knee; a plaster boot and bar splint was applied in osteoporotic cases. Knee range of motion exercises commenced on postoperative day 2, with isometric quadriceps, gluteal, and ankle pump exercises initiated within the first week. Toe-touch weight-bearing was allowed after approximately one week as pain permitted. Partial weight-bearing with a walker commenced at 6-8 weeks, progressing to full weight-bearing with walker support between 8-12 weeks, and eventually to independent full weight-bearing after radiological union confirmation. A structured follow-up protocol included assessments at 2 weeks, 1 month, 6-8 weeks, 8-12 weeks, 12-16 weeks, 16-20 weeks, 6 months, and 12 months. Range of motion and strengthening exercises were progressively advanced at each visit, including wall slides, heel raises, bridging, step exercises, gait training, and treadmill walking as tolerated.

Outcome measures

Functional outcome was assessed using the Harris Hip Score (HHS) [14] at 1, 3, 6, and 12 months, categorized as excellent (90-100), good (80-89), fair (70-79), and poor (<70). Pain was assessed using the Visual Analogue Scale (VAS, 0-10) at the same time points. Radiological outcome was evaluated using anteroposterior, lateral, and frog-leg lateral radiographs. Union was defined by bridging trabeculae across the fracture site, absence of progressive varus or translation, and pain-free full weight-bearing [15]. Fixation failure was defined as displacement >10 mm, progressive varus angulation, >5% change in screw axis on serial radiographs, >20 mm posterior translation, or femoral head perforation. Complications, including non-union, AVN, infection, implant failure, intraoperative hardware breakage, and screw backout, were recorded. Pauwels type-wise union rates were analyzed.

Statistical analysis

Data were analyzed using IBM SPSS Statistics for Windows, Version 26 (Released 2018; IBM Corp., Armonk, NY, USA). Continuous variables were expressed as mean ± standard deviation and categorical variables as frequencies, percentages, and 95% confidence intervals (Wilson score method). Changes in HHS and VAS between one month and subsequent follow-ups were analyzed using paired t-tests. Comparisons of continuous radiological parameters across Pauwels fracture types were performed using one-way analysis of variance (ANOVA). Categorical subgroup comparisons were evaluated using Fisher-family exact methods: the Freeman-Halton extension of Fisher's exact test was used for 2 × 3 contingency tables, and the mid-p corrected Fisher's exact test was used for sparse single-event comparisons. A p-value < 0.05 was considered statistically significant.

## Results

Baseline characteristics

The mean age was 50.6 years (range 40-60), with 16 of 25 patients (64.0%) being male and 9 of 25 (36.0%) being female. Regarding fracture laterality, 16 of 25 fractures (64.0%) involved the right hip. Falls on the ground accounted for 16 of 25 injuries (64.0%), followed by assault (5 of 25, 20.0%) and road traffic accidents (4 of 25, 16.0%). Distribution by Pauwels classification was as follows: type 1 (4 of 25, 16.0%), type 2 (12 of 25, 48.0%), and type 3 (9 of 25, 36.0%) (Table 1).

**Table 1:** Baseline demographic and injury characteristics of study participants (n = 25) Data are presented as n (%) for categorical variables and mean (range) for continuous variables.

Variable	Category/Unit	Value
Age (years)	Mean (range)	50.6 (40-60)
Age distribution	40-45 years	7 (28.0%)
	46-50 years	7 (28.0%)
	51-55 years	4 (16.0%)
	56-60 years	7 (28.0%)
Gender	Male	16 (64.0%)
	Female	9 (36.0%)
Side involved	Right	16 (64.0%)
	Left	9 (36.0%)
Mode of trauma	Fall on ground	16 (64.0%)
	Assault	5 (20.0%)
	Road traffic accident	4 (16.0%)
Pauwels type	Type 1	4 (16.0%)
	Type 2	12 (48.0%)
	Type 3	9 (36.0%)

Operative details

Mean intraoperative blood loss was 44 mL, with 20 of 25 patients (80.0%) experiencing blood loss of 30-50 mL. Mean operative time was 58.8 minutes, with 19 of 25 procedures (76.0%) completed within 30-60 minutes (Table 2).

**Table 2:** Operative details of calcar buttress fixation procedures (n = 25) Data are presented as n (%) for categorical variables and mean for continuous variables.

Variable	Category/Unit	Value
Intraoperative blood loss	Mean (mL)	44
Blood loss distribution	30-50 mL	20 (80.0%)
	51-70 mL	4 (16.0%)
	71-90 mL	1 (4.0%)
Operative time	Mean (minutes)	58.8
Operative time distribution	30-60 minutes	19 (76.0%)
	61-90 minutes	2 (8.0%)
	91-120 minutes	4 (16.0%)

Functional outcomes

Mean HHS improved significantly over time: 72.72 ± 7.22 at one month, 79.92 ± 6.19 at three months (p < 0.001), 84.64 ± 7.51 at six months (p < 0.001), and 87.84 ± 7.30 at 12 months (p < 0.001 compared with one month). At the 12-month follow-up, 14 of 25 patients (56.0%) achieved excellent HHS (90-100), 7 of 25 (28.0%) achieved good HHS (80-89), and 4 of 25 (16.0%) achieved fair HHS (70-79); none had poor outcomes. Paired t-tests confirmed statistically significant improvements at each follow-up interval (Table 3).

**Table 3:** Longitudinal functional and pain outcomes among study subjects (n = 25) Data are presented as mean ± SD. t-values were derived from paired t-test analysis. *p < 0.05 was considered statistically significant.

Outcome	Time Point	Mean ± SD	Comparison	t-value	p-value
Harris Hip Score	1 month	72.72 ± 7.22	Reference	-	-
	3 months	79.92 ± 6.19	3 vs 1 month	-8.12	<0.001*
	6 months	84.64 ± 7.51	6 vs 1 month	-12.36	<0.001*
	12 months	87.84 ± 7.30	12 vs 1 month	-14.96	<0.001*
Visual Analogue Scale	1 month	8.04 ± 0.74	Reference	-	-
	3 months	6.24 ± 0.93	3 vs 1 month	12.73	<0.001*
	6 months	4.28 ± 1.06	6 vs 1 month	21.38	<0.001*
	12 months	2.68 ± 1.38	12 vs 1 month	21.37	<0.001*

Pain outcomes

Mean VAS scores decreased significantly from 8.04 ± 0.74 at one month to 6.24 ± 0.93 at three months, 4.28 ± 1.06 at six months, and 2.68 ± 1.38 at 12 months (all p < 0.001 versus one month). Paired t-tests demonstrated highly significant pain reduction at all follow-up points (Table 3).

Radiological outcomes

Overall radiological union at 12 months was achieved in 24 of 25 patients (96.0%; 95% CI: 80.5%-99.3%). Union rates by Pauwels classification were as follows: type 1, 4/4 (100.0%; 95% CI: 51.0%-100.0%); type 2, 11/12 (91.7%; 95% CI: 64.6%-98.5%); and type 3, 9/9 (100.0%; 95% CI: 70.1%-100.0%). Freeman-Halton extension of Fisher's exact test showed no statistically significant difference in union rates across Pauwels types (p = 0.57), although this analysis was limited by very small subgroup sizes and sparse cell counts (minimum expected cell count < 1), which substantially reduced the reliability of formal hypothesis testing (Table 4).

**Table 4:** Radiological outcomes and complications stratified by Pauwels type (n = 25) Data are presented as n (%) (95% CI). p < 0.05 was considered statistically significant. FH-FET: Freeman-Halton extension of Fisher's exact test (2 × 3 table); Mid-p FET: mid-p corrected Fisher's exact test (2 × 2, T2 vs. pooled T1 + T3); NS: not significant

Variable	Overall (n = 25)	Pauwels Type 1 (n = 4)	Pauwels Type 2 (n = 12)	Pauwels Type 3 (n = 9)	Test	p-value
Union at 12 months	24 (96.0%) (80.5-99.3%)	4 (100.0%) (51.0-100.0%)	11 (91.7%) (64.6-98.5%)	9 (100.0%) (70.1-100.0%)	FH-FET	0.57 (NS)
AVN	1 (4.0%) (0.7-19.5%)	0 (0.0%)	1 (8.3%) (1.5-35.4%)	0 (0.0%)	Mid-p FET	NS (mid-p = 0.24)
Non-union	1 (4.0%) (0.7-19.5%)	0 (0.0%)	1 (8.3%) (1.5-35.4%)	0 (0.0%)	Mid-p FET	NS (mid-p = 0.24)
Deep infection	1 (4.0%) (0.7-19.5%)	0 (0.0%)	1 (8.3%) (1.5-35.4%)	0 (0.0%)	Mid-p FET	NS (mid-p = 0.24)
Screw backout	4 (16.0%) (6.4-34.7%)	0 (0.0%)	2 (16.7%) (4.7-44.8%)	2 (22.2%) (6.3-54.7%)	FH-FET	NS (p = 0.60)
Intraoperative drill bit breakage	1 (4.0%) (0.7-19.5%)	0 (0.0%)	1 (8.3%) (1.5-35.4%)	0 (0.0%)	Mid-p FET	NS (mid-p = 0.24)
Any complication	8 (32.0%) (17.2-51.6%)	0 (0.0%)	4 (33.3%) (13.8-60.9%)	4 (44.4%) (18.9-73.3%)	FH-FET	0.28 (NS)

Complications

Complications occurred in 8 of 25 patients (32.0%; 95% CI: 17.2%-51.6%). These included avascular necrosis (AVN) in 1/25 (4.0%), non-union in 1/25 (4.0%), deep infection requiring implant removal in 1/25 (4.0%), intraoperative drill bit breakage in 1/25 (4.0%), and screw backout in 4/25 (16.0%). The non-union occurred in a Pauwels type 2 fracture, while AVN also arose in the type 2 subgroup; whether these represent the same patient requires confirmation from individual patient records. No fixation failures, such as cut-out or femoral head perforation, were recorded (Table 4).

Radiological measurements 

Mean femoral neck shortening at 12 months was 5.3 ± 1.4 mm overall, with progressive shortening observed from 6 months (4.5 ± 1.4 mm) to 12 months (5.3 ± 1.4 mm). Pauwels type 3 fractures demonstrated the greatest shortening (6.5 ± 1.0 mm), compared to type 1 (3.5 ± 0.5 mm) and type 2 (4.9 ± 1.0 mm), with a statistically significant difference across groups (one-way ANOVA, p < 0.001) (Table 5).

**Table 5:** Radiological measurements at final follow-up (n = 25) One-way ANOVA was used for continuous variables; the Freeman-Halton extension of Fisher’s exact test was used for categorical variables across Pauwels types. *p < 0.05 was considered statistically significant. Values are presented as mean ± SD or n (%) (95% CI). GAI: Garden Alignment Index; NSA: Neck-Shaft Angle; FN: Femoral Neck

Radiological Parameter	Overall (n=25)	Pauwels Type 1 (n = 4)	Pauwels Type 2 (n = 12)	Pauwels Type 3 (n = 9)	p-value
Post-op GAI - AP view (°), Mean ± SD	157.3 ± 5.6	164.9 ± 2.1	158.0 ± 3.9	153.0 ± 4.6	0.002*
Acceptable reduction (GAI 155-180°), n (%) (95% CI)	19/25 (76.0%) (56.5-88.5%)	4/4 (100.0%) (51.0-100.0%)	9/12 (75.0%) (46.8-91.1%)	6/9 (66.7%) (35.4-87.9%)	0.31 (NS)
Post-op GAI - Lateral view (°), Mean ± SD	167.8 ± 4.9	172.0 ± 2.1	169.1 ± 4.3	164.2 ± 4.4	0.014*
Acceptable reduction (GAI 155-180°), n (%) (95% CI)	25/25 (100.0%) (86.8-100.0%)	4/4 (100.0%)	12/12 (100.0%)	9/9 (100.0%)	-
Neck-shaft angle at union (°), Mean ± SD	128.4 ± 4.9	130.9 ± 1.8	129.4 ± 3.6	125.8 ± 6.4	0.09 (NS)
NSA within 125°-135° (physiological), n (%) (95% CI)	20/25 (80.0%) (60.9-91.1%)	4/4 (100.0%)	10/12 (83.3%) (55.2-95.3%)	6/9 (66.7%) (35.4-87.9%)	0.28 (NS)
FN shortening at 6 months (mm), Mean ± SD	4.5 ± 1.4	3.0 ± 0.4	4.1 ± 1.1	5.8 ± 1.0	<0.001*
FN shortening at 12 months (mm), Mean ± SD	5.3 ± 1.4	3.5 ± 0.5	4.9 ± 1.0	6.5 ± 1.0	<0.001*
FN shortening >5 mm at 12 months, n (%) (95% CI)	13/25 (52.0%) (33.5-70.0%)	0/4 (0.0%) (0.0-49.0%)	5/12 (41.7%) (19.3-68.0%)	8/9 (88.9%) (56.5-98.0%)	0.004*
Radiological union at 12 months, n (%) (95% CI)	24/25 (96.0%) (80.5-99.3%)	4/4 (100.0%) (51.0-100.0%)	11/12 (91.7%) (64.6-98.5%)	9/9 (100.0%) (70.1-100.0%)	0.57 (NS)

Femoral neck shortening >5 mm at 12 months occurred in 13 of 25 patients (52.0%) and was associated with inferior functional outcomes: all patients with shortening >5 mm fell in the good or fair HHS category, while no patient with excellent HHS had shortening exceeding 5 mm (Table 6).

**Table 6:** Femoral neck shortening correlated with functional outcome (HHS at 12 months) HHS: Harris Hip Score

HHS Category at 12 Months	n	Union Status	Mean FN Shortening at 12 m (mm)	FNS >5 mm, n (%)
Excellent (90-100)	14	14/14 united	4.5	0/14 (0.0%)
Good (80-89)	7	7/7 united	5.8	4/7 (57.1%)
Fair (70-79)	4	3/4 united (1 non-union)	6.9	4/4 (100.0%)
Poor (<70)	0	-	-	-
Overall	25	24/25 (96.0%)	5.3 ± 1.4	13/25 (52.0%)

## Discussion

This prospective study evaluated calcar buttress BDSF fixation using a locking compression screw system in adults aged 40-60 years with intracapsular femoral neck fractures and demonstrated a high overall union rate of 24/25 (96.0%) with predominantly excellent or good functional outcomes at 12 months. Mean HHS improved from 72.72 at 1 month to 87.84 at 12 months, and mean VAS decreased from 8.04 to 2.68, indicating substantial and clinically meaningful recovery of hip function and pain relief. These findings support calcar-buttressed BDSF fixation as an effective head-preserving option in this intermediate age group [3,16].

Intracapsular femoral neck fractures have historically been termed “unsolved fractures” due to high rates of non-union (10%-30%) and AVN (10%-33%) despite advances in implant design [6,7]. The rates of non-union (1/25, 4.0%) and AVN (1/25, 4.0%) in this series compare favorably with conventional CCS literature, although the 12-month follow-up mandates cautious interpretation, as late AVN may manifest beyond one year [17].

The BDSF concept addresses mechanical shortcomings of parallel CCS constructs by positioning screws in two planes with dual cortical support, transforming the construct into a vertical beam [8]. Filipov reported union rates of approximately 98%-99% and mean HHS in the mid-80s using BDSF [9]. The current study's mean HHS of 87.8 is broadly consistent with these reports, despite a younger cohort, suggesting that biomechanical advantages of calcar-buttress fixation translate into good functional recovery in active middle-aged adults.

Several comparative studies contextualize these findings. Filipov and Gueorguiev demonstrated in cadaveric biomechanical studies that BDSF constructs exhibited superior resistance to varus collapse and rotation compared with conventional CCS patterns, particularly in osteoporotic bone [18]. Shi et al. reported that BDSF yielded higher union rates and better functional outcomes than conventional CCS [10]. However, finite element analysis by Xia et al. suggested that CCS combined with medial buttress plating demonstrated superior biomechanical performance compared with BDSF alone in vertical femoral neck fractures [11].

An important implication of the revised radiological dataset is that union rates were uniformly high across all Pauwels fracture types (type 1: 100%, type 2: 91.7%, type 3: 100%), with no statistically significant intergroup difference (p = 0.57). This is consistent with the biomechanical principle that more vertical fracture orientations are associated with greater shear forces at the fracture site, driving ongoing collapse during the healing phase even when fixation is maintained, and union is ultimately achieved [19].

Femoral neck shortening greater than 5 mm at 12 months was observed in 13/25 patients (52.0%) overall and in 8/9 type three patients (88.9%) and was systematically associated with inferior functional categories. No patient achieving excellent HHS had shortening >5 mm, while all patients in the fair HHS category had shortening exceeding this threshold. This aligns with published evidence demonstrating that femoral neck shortening impairs abductor mechanism function, alters gait mechanics, and reduces quality of life following internal fixation. In this cohort, shortening is therefore a more sensitive indicator of post-surgical biomechanical compromise than union status alone, and its monitoring should be integrated into routine follow-up protocols [19].

An important observation is the 4 of 25 (16.0%) incidence of screw backout despite using a locking head compression screw system. Theoretical advantages of locking compression screws include angular stability and resistance to migration; however, screw backout still occurred, typically after weight-bearing initiation. This rate appears higher than that reported by Filipov using non-locking CCS in BDSF constructs [9]. These findings suggest that the locking mechanism does not fully compensate for factors such as bone quality, fracture geometry, or dynamic loading, and surgeons should continue to pay meticulous attention to reduction quality, screw trajectory, and postoperative loading protocols [20,21].

Limitations

This study carries a few limitations. First, the sample size is modest (n = 25), limiting statistical power for subgroup analyses. The mid-study interruption of implant supply under the Ayushman Bharat Yojana scheme represents a potential recruitment bias and is acknowledged as a study limitation. Second, the study is single-centre and non-comparative, so relative efficacy against alternative constructs cannot be established. Third, a 12-month follow-up precludes evaluation of late AVN, post-traumatic osteoarthritis, or long-term implant survivorship. Fourth, the learning curve associated with this technique may have contributed to technical complications in early cases. Fifth, pre-operative functional scores (HHS and VAS) were not recorded; therefore, all longitudinal comparisons reflect improvement from one month post-operatively, not from baseline. This prevents quantification of the absolute treatment effect and is a recognised methodological limitation. Sixth, DEXA scanning was not uniformly available at our institution, so no bone mineral density or surrogate bone quality measure was available for study subjects. Seventh, the absence of routine cross-sectional imaging is another limitation, as CT or MRI could have better demonstrated subtle fracture features such as occult posterior comminution. Lastly, bone quality was not objectively measured, and unmeasured osteopenia or osteoporosis may have confounded fixation stability and shortening outcomes. Future research should focus on well-designed comparative trials incorporating longer follow-up, quantitative shortening measurement, and biomechanical analysis to refine operative indications and technique.

## Conclusions

Calcar buttress BDSF fixation using a locking compression screw system appears to be a head-preserving fixation technique with encouraging early results for intracapsular femoral neck fractures in adults aged 40-60 years, achieving a high overall union rate and favorable 12-month functional outcomes. However, Pauwels type III fractures showed greater femoral neck shortening, and shortening greater than 5 mm was associated with inferior functional recovery, indicating that post-healing alignment may be a more informative endpoint than union alone. Given the small sample size and wide subgroup confidence intervals, these findings should be interpreted cautiously and confirmed in larger comparative studies.

## References

[1] Rahim KA, Arifeen KM, Begum R (2025). Evaluation of cemented bipolar hemiarthroplasty to assess functional outcome of displaced femoral neck fractures in elderly osteoporotic patients. J Ortho Sports Med.

[2] Czerwonka N, Desai SS, Gupta P, Shah RP, Geller JA, Cooper HJ, Neuwirth AL (2024). Perioperative outcomes of intramedullary nail vs hemiarthroplasty vs total hip arthroplasty for intertrochanteric fracture: an analysis of 31,519 cases. Arthroplast Today.

[3] Cai L, Zheng W, Chen C, Hu W, Chen H, Wang T (2024). Comparison of young femoral neck fractures treated by femoral neck system, multiple cancellous screws and dynamic hip screws: a retrospectively comparison study. BMC Musculoskelet Disord.

[4] Yang J, Zhou X, Li L (2021). Comparison of femoral neck system and inverted triangle cannulated screws fixations in treatment of Pauwels typle Ⅲ femoral neck fractures (Article in Chinese). Zhongguo Xiu Fu Chong Jian Wai Ke Za Zhi.

[5] Filipov O (2013). The method of biplane double-supported screw fixation (BDSF) at femoral neck fractures - principle and clinical outcomes. J IMAB.

[6] Filipov O, Stoffel K, Gueorguiev B, Sommer C (2017). Femoral neck fracture osteosynthesis by the biplane double-supported screw fixation method (BDSF) reduces the risk of fixation failure: clinical outcomes in 207 patients. Arch Orthop Trauma Surg.

[7] Filipov OB (2019). Biplane double-supported screw fixation of femoral neck fractures: surgical technique and surgical notes. J Am Acad Orthop Surg.

[8] Rajnish RK, Srivastava A, Rathod PM (2022). Does the femoral neck system provide better outcomes compared to cannulated screws fixation for the management of femoral neck fracture in young adults? A systematic review of literature and meta-analysis. J Orthop.

[9] Filipov O (2011). Biplane double-supported screw fixation (F-technique): a method of screw fixation at osteoporotic fractures of the femoral neck. Eur J Orthop Surg Traumatol.

[10] Shi XC, Chu ZT, Yuan YW, Xia XL, Li WL, Wang CB, Hu JJ (2025). Femoral calcar double-supported screw fixation enhances biomechanical stability in Pauwels type III femoral neck fractures: a comparative biomechanical study. Am J Transl Res.

[11] Xia Y, Zhang W, Hu H, Yan L, Zhan S, Wang J (2021). Biomechanical study of two alternative methods for the treatment of vertical femoral neck fractures - a finite element analysis. Comput Methods Programs Biomed.

[12] Bartonícek J (2001). Pauwels' classification of femoral neck fractures: correct interpretation of the original. J Orthop Trauma.

[13] Garden RS (1961). Low-angle fixation in fractures of the femoral neck. J Bone Joint Surg Br.

[14] Harris WH (1969). Traumatic arthritis of the hip after dislocation and acetabular fractures: treatment by mold arthroplasty. An end-result study using a new method of result evaluation. J Bone Joint Surg Am.

[15] Zahid M, Bin Sabir A, Asif N, Julfiqar M, Khan AQ, Ahmad S, Siddiqui YS (2012). Fixation using cannulated screws and fibular strut grafts for fresh femoral neck fractures with posterior comminution. J Orthop Surg (Hong Kong).

[16] Rogmark C, Johnell O (2006). Primary arthroplasty is better than internal fixation of displaced femoral neck fractures: a meta-analysis of 14 randomized studies with 2,289 patients. Acta Orthop.

[17] Jilani LZ, Abbas MB, Shaan ZH, Ahmad S, Abbas M (2023). Outcomes of BDSF technique for osteosynthesis of femoral neck fractures. Int J Burns Trauma.

[18] Filipov O, Gueorguiev B (2015). Unique stability of femoral neck fractures treated with the novel biplane double-supported screw fixation method: a biomechanical cadaver study. Injury.

[19] Lee DH, Kwon JH, Kim KC (2024). The effects and risk factors of femoral neck shortening after internal fixation of femoral neck fractures. Clin Orthop Surg.

[20] Slobogean GP, Stockton DJ, Zeng BF, Wang D, Ma B, Pollak AN (2017). Femoral neck shortening in adult patients under the age of 55 years is associated with worse functional outcomes: analysis of the prospective multi-center study of hip fracture outcomes in China (SHOC). Injury.

[21] Wang K, Lin D, Chen P (2023). Incidence and factors influencing neck shortening after screw fixation of femoral neck fractures with the femoral neck system. J Orthop Surg Res.

